# Supplementary material for: Trichromacy is insufficient for mate detection in a mimetic butterfly
Source: Commun Biol. 2025 Feb 6;8:189. doi: 10.1038/s42003-025-07472-7 (PMC11802900; doi:10.1038/s42003-025-07472-7)
Supplement: Supplementary file 1 — Supplementary Information [file 42003_2025_7472_MOESM1_ESM.pdf]

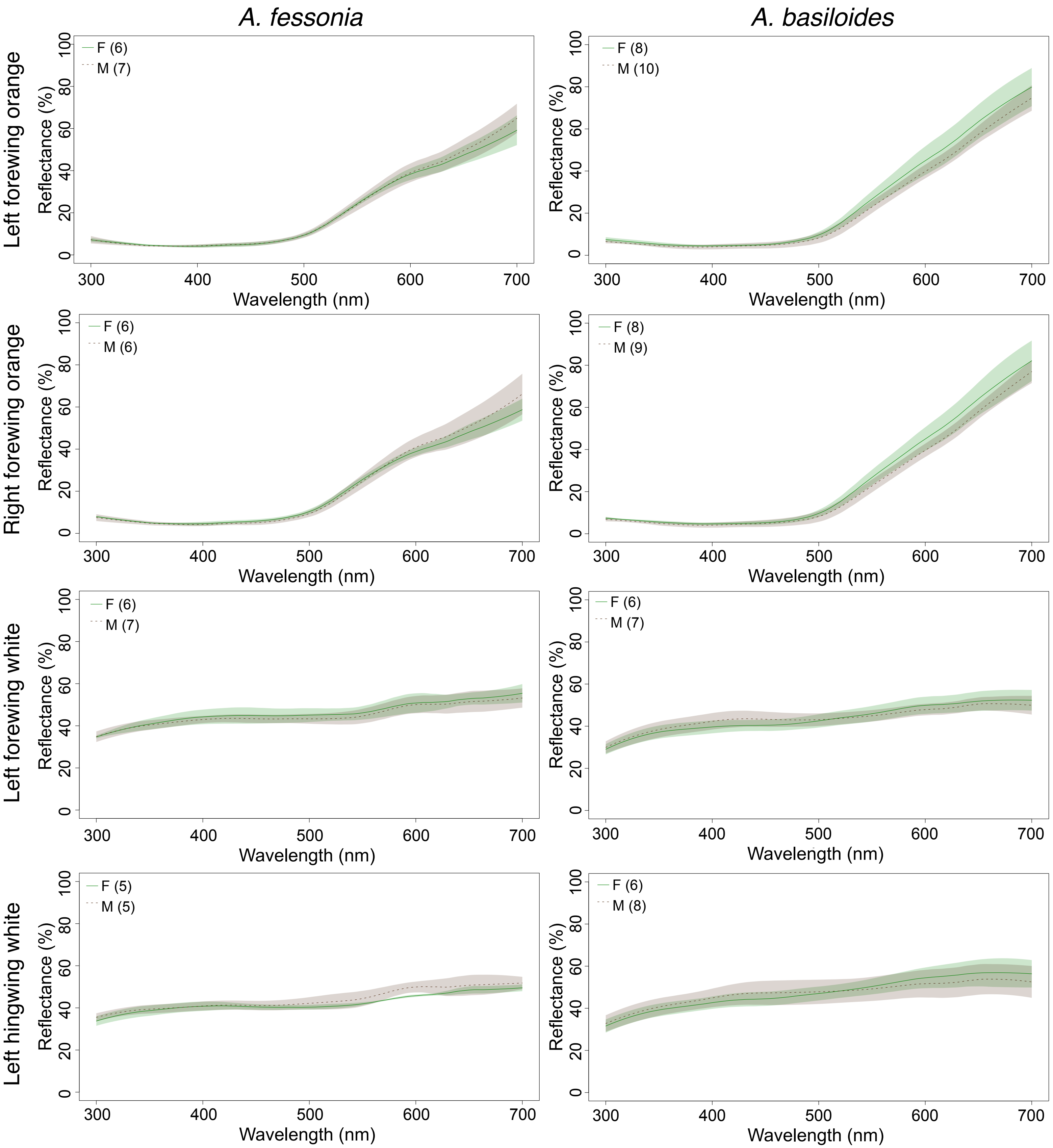

Supplementary Figure 1. Reflectance spectra of orange and white dorsal wing colors of male and female *Adelpha fessonia* and *A. basiloides*. The number of individual butterflies measured for each comparison are indicated as numbers in parentheses with *A. fessonia* on the left and *A. basiloides* on the right.

Supplementary Information for Dang et al.

Supplementary Figure 1. Reflectance spectra of orange and white dorsal wing colors of male and female *Adelpha fessonia* and *A. basiloides.* The number of individual butterflies measured for each comparison are indicated as numbers in parentheses with *A. fessonia* on the left and *A. basiloides* on the right.

Supplementary Data 1. Specimen collecting locality, sex, sequencing method, and opsin Genbank accession numbers.

Supplementary Data 2. RNA-seq library assembly stats.

Supplementary Data 3. Specimen collecting locality and sex for wing reflectance measurements.
