# Supplementary material for: Trichromacy is insufficient for mate detection in a mimetic butterfly
Source: Commun Biol. 2025 Feb 6;8:189. doi: 10.1038/s42003-025-07472-7 (PMC11802900; doi:10.1038/s42003-025-07472-7)
Supplement: Supplementary file 2 — Description of Additional Supplementary File [file 42003_2025_7472_MOESM2_ESM.pdf]

Description Of Additional Supplementary File

File name: Supplementary Data 1.
Description: Specimen collecting locality, sex, sequencing method, and opsin Genbank accession numbers.

File name: Supplementary Data 2.
Description: RNA-seq library assembly stats.

File name: Supplementary Data 3.
Description: Specimen collecting locality and sex for wing reflectance measurements.
